# Morphological characteristics of cartilage-bone transitional structures in the human knee joint and CAD design of an osteochondral scaffold

**DOI:** 10.1186/s12938-016-0200-3

**Published:** 2016-07-14

**Authors:** Weiguo Bian, Qin Lian, Dichen Li, Jin Wang, Weijie Zhang, Zhongmin Jin, Yusheng Qiu

**Affiliations:** The First Affiliated Hospital of Xi’an Jiaotong University, Xi’an, 710061 Shaanxi China; State Key Lab for Manufacturing System Engineering, Xi’an Jiaotong University, Xi’an, 710049 Shaanxi China; The Second Affiliated Hospital of Medical College, Xi’an Jiaotong University, Xi’an, 710028 Shaanxi China

**Keywords:** Cartilage-bone transitional structure, Cartilage-bone interface, Human knee joint, Tissue engineering, Computational modeling, Osteochondral scaffold

## Abstract

**Background:**

There is a lack of understanding of the morphological characteristics of the cartilage-bone interface. Materials that are currently being used in tissue engineering do not adequately support the regeneration of bone and cartilage tissues. The present study aimed to explore the morphological characteristics of cartilage-bone transitional structures in the human knee joint and to design a biomimetic osteochondral scaffold based on morphological data.

**Methods:**

Histology, micro-computed tomography (micro-CT), and scanning electron microscopy (SEM) were used to investigate the microstructure of the cartilage-bone transitional structures. Morphological characteristics and their distribution were obtained and summarized into a biomimetic design. A three-dimensional model of a biomimetic osteochondral scaffold was CAD designed. A prototype of the resulting subchondral bone scaffold was constructed by stereolithography using resin.

**Results:**

Micro-CT revealed that subchondral bone presented a gradually changing structure from the subchondral to spongy bone tissue. The subchondral bone plate was more compact with ~20 % porosity compared with ~60 % porosity for the spongy bone. Histology and SEM showed that cartilage was stabilized on the subchondral bone plate by conjunctions, imbedding, interlocking, and binding forces generated by collagen fibers. Some scattered defects allow blood vessel invasion and nutritional supply.

**Conclusions:**

The subchondral bone plate is not an intact plate between the cartilage and bone cavity, and some scattered defects exist that allow blood vessel invasion and nutritional supply. This characteristic was used to design an osteochondral scaffold. This could be used to construct an osteochondral complex that is similar to native bones.

## Background

Over the last decade, many three-dimensional (3D) osteochondral scaffolds have been designed using a number of heterogeneous materials. These osteochondral scaffolds have the potential to be used for repairs of the knee and for the treatment of osteoarthritis [1, 2], but the heterogeneous materials do not adequately support the formation of hyaline cartilage in large animal models because of unsuitable microarchitecture, unstable conjunctions, poor mechanical properties, and/or improper blood flow [1–3]. Given the importance of this structure-to-function relationship, a major objective of bone scaffold design is to provide an optimal environment where regeneration of bones and cartilage tissues can take place [3]. In the existing designs (direct cross, radial, and casual structures), only pore size and porosity vary, which cannot satisfy the different requirements of bone and cartilage regeneration [4–11].

Therefore, the solution could be to design and build an osteochondral scaffold with a true biomimetic microarchitecture reflecting the morphology of the natural osteochondral complex, which is made of bone, cartilage, and their transitional structures. The transitional structures including the calcified cartilage zone (CCZ) and the subchondral bone plate provide the attachment of cartilage to bone, allow force transmission across the joint, and limit the diffusion from the subchondral bone [12, 13].

Wang et al. [14] used histomorphometric methods to explore the microstructures of the CCZ and to generate a 3D model. They discovered two junctional interfaces that connected the CCZ to uncalcified cartilage and subchondral bone [14]. The upper interface between the CCZ and the hyaline cartilage is called the tidemark, while the lower interface between the CCZ and the subchondral bone is called the cement line [14–16]. The subchondral bone area can be divided as the subchondral bone plate and trabecular bone. The subchondral bone plate is the bone layer separating the calcified cartilage from bone marrow spaces [17], and provides most of the cushioning for the joint. In addition, the subchondral bone maintains an incongruent joint surface, which is ideal for maintaining a physiological joint environment [17]. Pathological changes in the subchondral bone plate such as thickening, sclerosis, or osteoporosis occur at the onset of osteoarthritis, even before the pathological progression of articular cartilage [18, 19].

Many studies have focused on osteochondral complex structures and their pathological changes in joint abnormalities [4–11], but few hierarchically engineered models have been presented based on the exact morphological characteristics of bone, cartilage, and their transitional structures. Although the morphological map of bone and cartilage is well known, a crucial obstacle for building a model of cartilage-bone transitional structures is the complexity and diversity of model of cartilage-bone transitional structures.

Therefore, the present study aimed to explore the morphological characteristics of cartilage-bone transitional structures, specifically the micro-architectures and conjunctions of the osteochondral complex in the human knee joint, and to design a biomimetic osteochondral scaffold based on these morphological data.

## Methods

### Specimens

Eight human knees were extracted from four cadavers (#1: 25 years-old male; #2: 39 years-old male; #3: 41 years-old female; #4: 27 years-old female); they were fixed and preserved in formalin. These cadavers were never frozen. The specimens were investigated at the Department of Anatomy of Xi’an Jiao Tong University. Samples were from individuals who donated their corpse to science after their death. This study was approved by the Anatomic Gift Program of the Xi’an Jiao Tong University (No. 2012-211). All specimens had no cartilage defect or pathological change indicative of osteoarthritis (International Cartilage Repair Society [20] Grade 0).

### Micro-computed tomography (micro-CT)

**Fig. 1:**
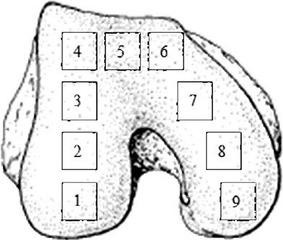
Schematic layout of samples taken from the distal femur. *1* back of the lateral condyle; *2* middle of the lateral condyle; *3* front of the lateral condyle; *4* lateral part of the femoral trochlea; *5* middle of the femoral trochlea; *6* medial part of the femoral trochlea; *7* front of the medial condyle; *8* middle of the medial condyle; and *9* back of the medial condyle

**Fig. 2:**
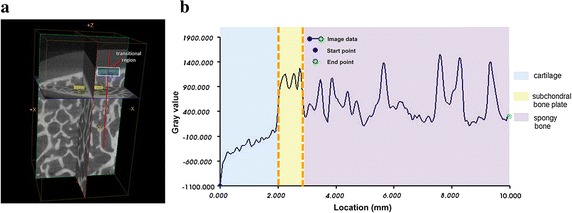
Changes of gray value along the line from cartilage to spongy bone in the three-dimensional model generated by micro-computed tomography. **a** Scanning picture of sample excised from the *middle* of the medial condyle position (Fig. 1) of cadaveric *right* *knee*. The *yellow* *boxes* are examples of volume of interests; the *white* *box* is example of transitional region; the *red* *line* indicates the direction of gray value detection. **b** Gray value variation along the line from cartilage to spongy bone. The *red* *line* in (**a**) corresponds to the *yellow* *area* in (**b**). The distance from the surface of cartilage to the subchondral plate below was about 2.3 mm

Two samples were excised from the middle of the medial condyle of two cadaveric right knees as cubes (10 × 10 × 10 mm) (Fig. 1) and were placed in the sample holder of the micro-CT scanner (eXplore Locus SP, GE Healthcare, USA). The micro-CT experiment was performed to confirm the SEM and histology observations described below. The following scanning parameters were used: 80 kV; 80 µA; 6.4 W; exposure of 3 s; filter of 0.02 mm Al; tube volume: 44 ml; slice thickness: 21 μm; and scan time: 150 min. A volume of interest (VOI) was first selected, and the 3D structure of the VOI was designed in the scanner’s MicroView software. To get accurate data, the samples were revised using the Hounsfield calibration (GE Healthcare, USA). The reconstruction method was filter-back projection and no post-processing was applied. After calibration, the bone parameters of the selected VOI were measured using the scanner’s MicroView software. In the present study, 29 VOIs from the transitional region between the cartilage and bone to the spongy bone were selected as the measurement points along the line that was vertical to the interface (Fig. 2a). The bone mineral density (BMD), tissue mineral density (TMD), bone mineral content (BMC), tissue mineral content (TMC), and bone volume fraction (BVF) were measured and analyzed. These parameters are related by the following equations [21, 22]:

$$ BMD = {{BMC} \mathord{\left/ {\vphantom {{BMC} V}} \right. \kern-0pt} V}_{total} $$

$$ TMD = {{TMC} \mathord{\left/ {\vphantom {{TMC} V}} \right. \kern-0pt} V}_{bone} $$

$$ Porosity = 1 - BVF $$

where V is the volume.

### 3D model generation by micro-CT

The images were analyzed in terms of gray values. The gray value of water was calibrated as 0, the gray value of air was −1000, and the gray value of cortical bone was 2000 [23]. A line was drawn from the cartilage to the spongy bone in the 3D model (Fig. 2a), and the changes in gray values along the line were determined using the scanner’s MicroView software. Because the subchondral bone plate is curved, tracking was manually operated in order to ensure that measurements were mostly perpendicular. Combined with the length measurement model, the gray values were plotted and the graph was divided into three parts: the rising part is the cartilage, the plateau is the subchondral bone plate, and the fluctuating part is the spongy bone (Fig. 2b).

### Scanning electron microscopy (SEM) and digital micrography

Nine samples were excised from each cadaveric left knee as cubes (10 × 10 × 5 mm) from the regions shown in Fig. 1 and immediately oxidized with 30 % O_2_ for 24 h. The samples were placed in an ultrasonic bath for 1 h. The oxidation and washing process was repeated four times to remove cartilage. Two samples were excised from the middle of the medial condyle position of two cadaveric right knees as cubes (10 × 10 × 10 mm) (Fig. 1). All samples were placed in 2 % glutaraldehyde solution, dehydrated with graded ethanol, and dried using the critical point drying method. The dried samples were glued onto aluminum stubs, sputter-coated with gold, and examined using SEM (DJM-840, JEOL, Japan) at 20 kV. After SEM examination, samples were observed using digital micrography (keyence, Japan) and 3D micrographs were reconstructed. The smallest inscribing circle was drawn within the canals on SEM images using Adobe Photoshop CS5 (Adobe Systems, San Jose, CA, USA), and the final value was determined based on the scale bar.

### Histological evaluation

Four samples were excised from the middle of the lateral condyle position of four cadaveric right knees as cubes (10 × 10 × 5 mm) (Fig. 1). After fixation, samples were dehydrated using an ethanol gradient (50, 70, 95, and 100 %) and cleared using xylene. The samples were embedded with paraffin and sectioned (5 μm). The sections were stained with Safranin O/Fast Green (Sigma, USA) [24] and Masson trichrome [25]. The samples were analyzed using light microscopy (SP1600, Leica, Germany). All resections were performed by the same surgeon.

### 3D CAD model

Considering the requirements of osteochondral tissue engineering and the limits of actual construction techniques, a 3D model of the bone transitional phase was constructed using the Pro/ENGINEER software (PTC, Needham, USA) according to the morphological characteristics acquired from the observation of the natural osteochondral complex.

### Prototyping of the osteochondral scaffold

To determine if the resulting scaffold could be manufactured, a prototype of the resulting subchondral bone scaffold was constructed by stereolithography using resin, based on a previously published method [26].

## Results

### 3D model generated by micro-CT

The slow increase in gray value observed in the plot of the 3D cartilage model suggests that the cartilage closer to the bone had a high gray value (Fig. 2b). The plateau part of the plot indicated that the subchondral bone plate of the knee joint was more compact than spongy bone, but was not intact. The great fluctuation of the diagram reflected the trabecular bone and the pores in this region. The thickness of the cartilage in the subchondral bone region was about 2.3 mm (obtained from two specimens: 2.29 and 2.31 mm, respectively) (Fig. 2b).

### Characterization of bone and cartilage parameters

**Fig. 3:**
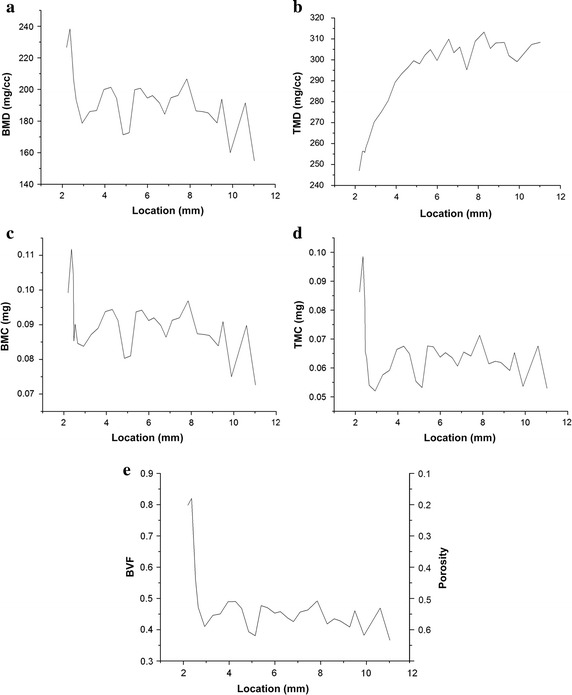
Diagram of variations in bone parameters along the line from cartilage to spongy bone. Bone parameters of the selected volume of interests (Fig. 2) were measured using micro-computed tomography the scanner’s MicroView software. **a** Bone mineral density *BMD*; **b** tissue mineral density *TMD*; **c** bone mineral content *BMC*; **d** tissue mineral content *TMC*; **e** bone volume fraction *BVF* and porosity. The distance from the surface of cartilage to the subchondral plate below was about 2.3 mm

The BMD, BMC, and TMC values were the highest around the subchondral bone region; the distance from the surface of cartilage to the subchondral plate below was about 2.3 mm (Fig. 3a, c, d), indicating that the mineral content in the subchondral bone plate was higher than in the trabecular bone. TMD value increased slowly as the location of VOI was moved away from the cartilage, with a maximal TMD value obtained at the plateau, which was 4.3 mm away from the cartilage surface (Fig. 3b). The trabecular bone had lower porosity and higher gray value than the subchondral bone plate. The BVF was higher in the subchondral bone plate than in the spongy bone, and changed greatly at 2.3 mm (Fig. 3e). The two levels of porosity distribution of the subchondral bone were as follows: about 20 % of porosity at 2.3 mm, corresponding to the subchondral bone plate; and about 60 % of porosity, above 2.3 mm, corresponding to spongy bone (Fig. 3e).

### Morphological characteristics of the cartilage-bone interface

**Fig. 4:**
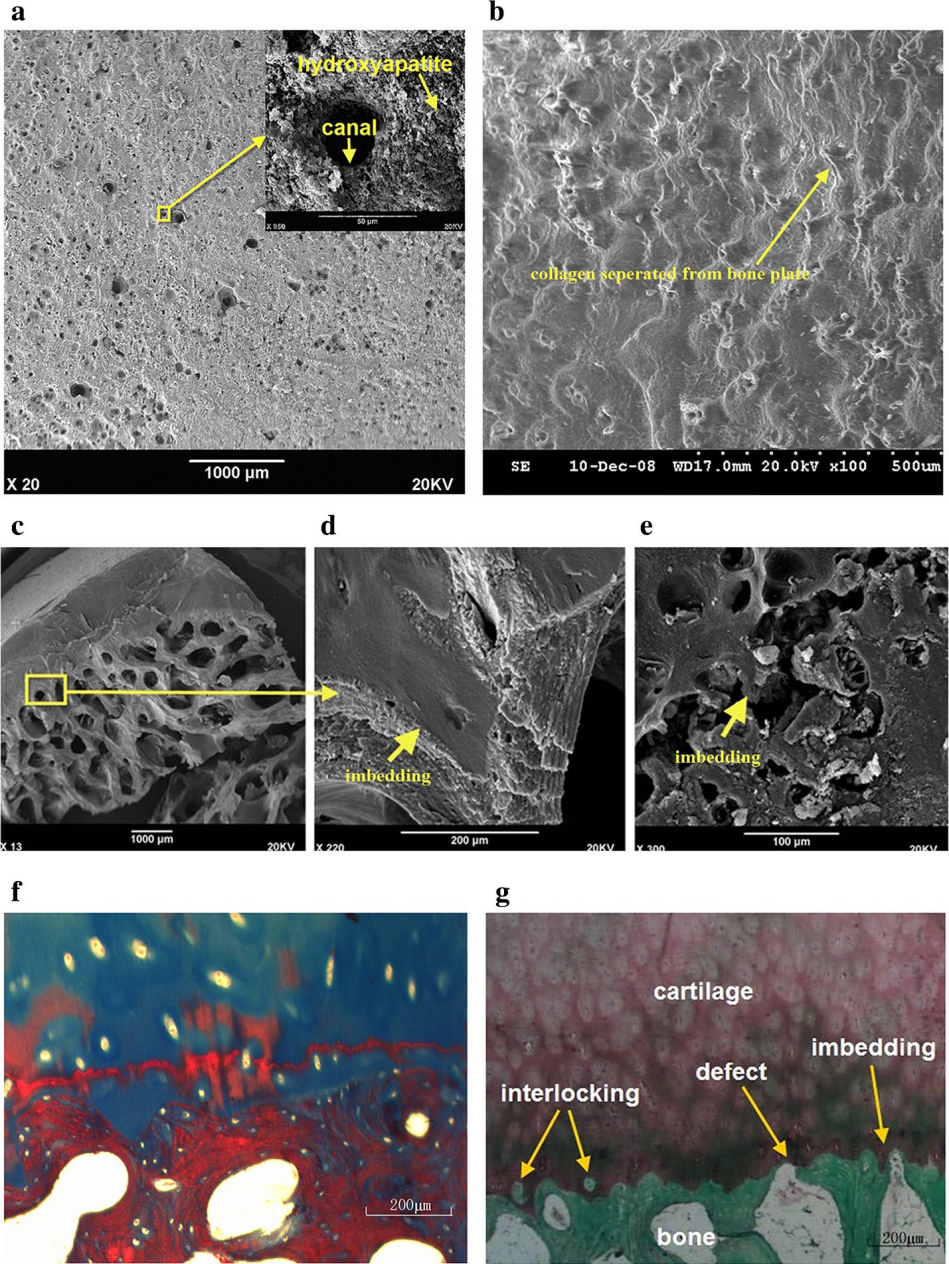
Micrographs were obtained using scanning electron microscopy *SEM* and histology. **a** The interface after cartilage removal from the subchondral bone plate using oxidation (*scale bar* 1000 μm). All collagen has been removed and the hydroxyapatite particles and canals formed by the cartilage are apparent. **b** The *upper surface* of the calcified cartilage zone after hyaline cartilage separation by ultrasonic shaking (*scale* *bar* 500 μm). Three-dimensional micrographs were reconstructed using digital microscopy. Micrographs were obtained using SEM. Collagen fibers separated from the bone plate could be observed. **c** Sectional view of the bone and cartilage without removing the cartilage from the bone. **d** Enlargement of 4C. The imbedding structure could be observed. **e** Sectional view of the bone after removing the cartilage. *Arrows* show the connection between bone and cartilage. Histological micrographs of Masson trichrome staining (**f**) and Safranin O/Fast* Green* staining (**g**) show the transitional structures between the subchondral bone and the cartilage (*scale bar* 200 μm): interlocking, defect, and imbedding

Projections of cartilage breaking into the subchondral bone plate formed a kind of connecting structure. After removing the cartilage, there were pore-like structures on the bone surface of the subchondral bone plate, which were called canals (Fig. 4a). This image suggests that there might be some differences between hard tissues and the upper surface of the CCZ after removing the hyaline cartilage by ultrasonic shaking (Fig. 4b). The diameter of the canals (pointed by arrows in Fig. 4a) was between 10 and 160 μm (Table 1). The overall area of the canals and hollows was 7 % of the entire measured surface (n = 36). The connection between bone and cartilage before (Fig. 4c, d) and after removing the cartilage (Fig. 4e) showed that the cartilage stretched out into the hollows or defects on the surface of the subchondral bone. Masson trichrome staining is shown in Fig. 4f. Safranin O/Fast Green staining also showed that the cartilage stretched out into the hollows or defects on the surface of the subchondral bone (Fig. 4g). Figure 4g also shows that the cartilage was stabilized on the subchondral bone plate by conjunctions, imbedding, interlocking, and binding forces generated by collagen fibers and the rough surface of the bone matrix. The subchondral bone plate was not an intact and smooth plate between the cartilage and bone cavity.

**Table 1:** Diameter distribution of canals on the subchondral bone surface

Regions (from Fig. 1)	Diameter distribution (µm)
Region 1	35.46 ± 13.90
Region 2	35.40 ± 14.66
Region 3	28.19 ± 7.48
Region 4	33.02 ± 9.59
Region 5	33.06 ± 11.92
Region 6	33.50 ± 10.50
Region 7	22.08 ± 9.68
Region 8	44.24 ± 19.73
Region 9	45.49 ± 15.37
Total (N = 1814)	34.08 ± 14.02

### Bio-inspired design

**Fig. 5:**
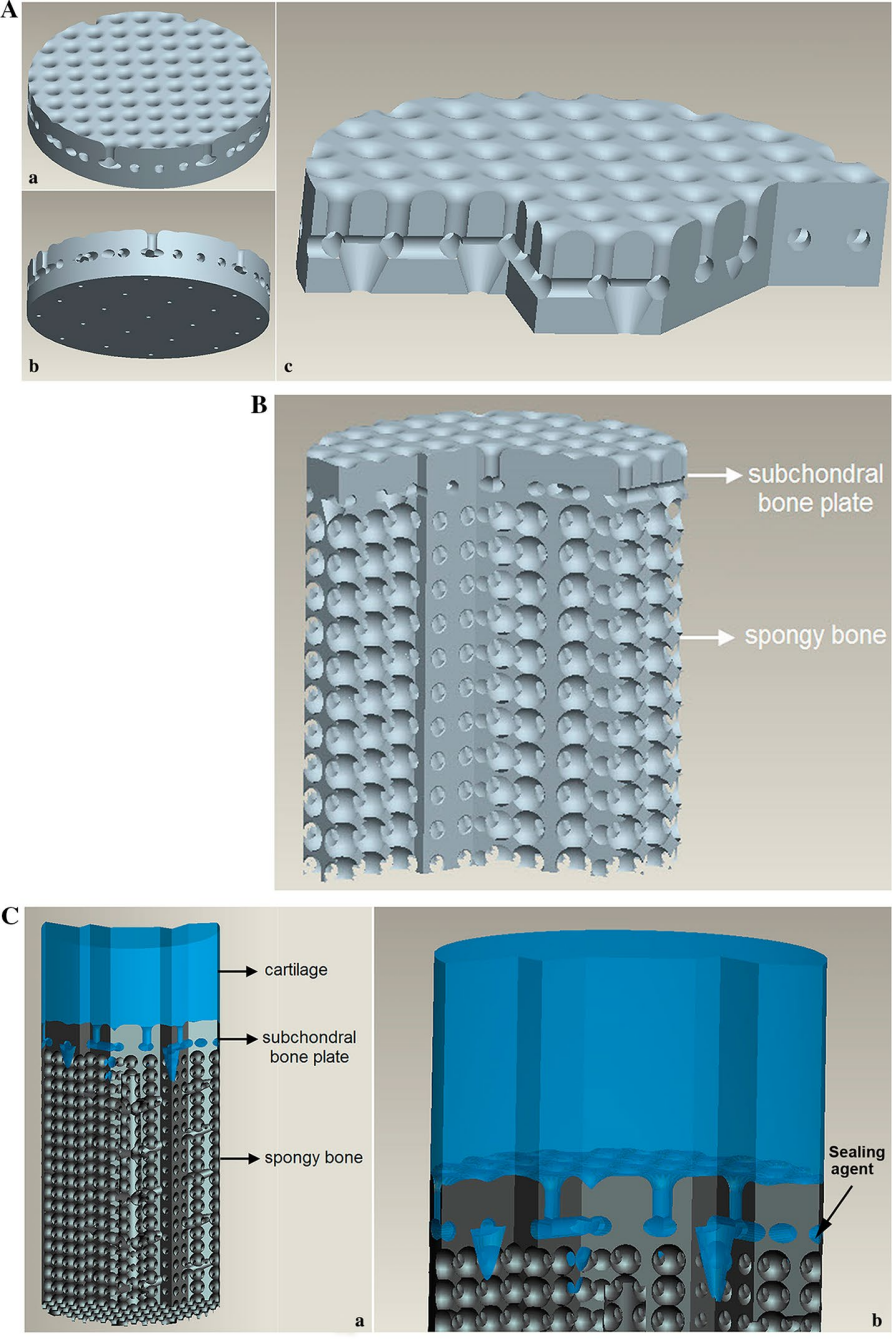
Bioinspired design of an osteochondral scaffold. **A** Engineered three-dimensional mold of the transitional phase with a biomimetic bonding structure and a potentially limited entrance for vascularization: *a* cartilage side, *b* bone side, and *c* the cross-section showing gomphosis, interlocking, and limited defects. **B** Engineered three-dimensional mold of the ceramic scaffold with an interconnected porous bone phase and biomimetic transitional phase. **C** Schematic bonding of the chondral phase and the ceramic phase by the biomimetic transitional structures: *a* cross section of the entire bioinspired osteochondral scaffold, *b* type-I collagen solution that is drawn into the bonding structures on the *upper side* of the ceramic phase. A sealing agent (*arrow*) was applied between the subchondral bone plate and cancellous bone to block some pores that were communicating with cancellous bone layer

A 3D CAD model of the osteochondral scaffold was designed based on the morphological characteristics of the natural osteochondral complex. The design of the cartilage and cancellous bone binding part was made from the subchondral bone plate data, and considering the manufacturing factors and limits. The characteristics of the scaffold were: porosity of 30 % (the subchondral bone plate had a porosity of 15–40 %; the median of 30 % was taken for the scaffold), 9.61 % pore area of the total surface area (which was observed on the entire subchondral bone plate), and pore diameter ratio of 35 % (pore diameter over the entire slice length) (Fig. 5Aa).

**Fig. 6:**
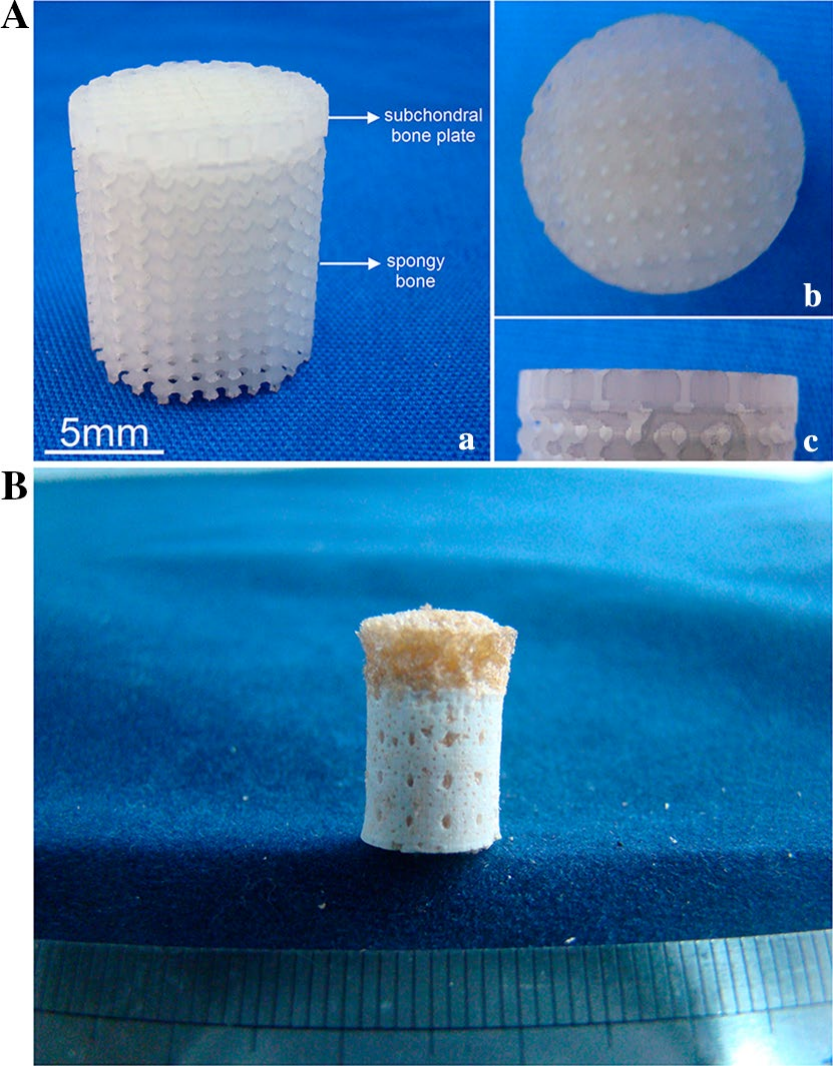
Final sample piece of biomimetic osteochondral scaffold. **A** The final osteochondral scaffold test piece comprised of the subchondral bone plate and spongy bone fabricated using stereolithography of resin (*a*), the surface of subchondral bone plate of the test piece (*b*), and the lateral aspect of subchondral bone plate of the test piece (*c*). **B** The final biomimetic osteochondral scaffold after the cartilage phase was added by gel casting

The design of the cancellous bone area was derived from the data portion of cancellous bone, but cell growth and the manufacturing process were considered in this design [26, 27], resulting in a porosity of bone phase of 65 %. Based on a previous study [27], the porous structure was designed to mimic the trabecular bone structure with the following features: 800 μm pore size, 400 μm interconnected pore size, and 100 % interconnected porosity. A sealing agent (type-I collagen solution) was applied to prevent blood vessel invasion (Fig. 5Cb). Some conical vents were designed to penetrate the sealing agent and to connect with the bone phase to simulate the imbedding and defects (Fig. 5Ab, c). Figure 5B shows the engineered three-dimensional mold of the ceramic scaffold with an interconnected porous bone phase and biomimetic transitional phase. Schematic bonding of the chondral phase and the ceramic phase by the biomimetic transitional structures is shown in Fig. 5C. Table 2 shows the comparative analysis of the morphological characteristics between natural osteochondral complex and the proposed scaffold for osteochondral tissue engineering. This design included the engineering issues that had to be taken into account. The growth of bone cells need a certain spatial structure of engineered scaffold such as a suitable pore size, porosity, and connectivity rate. When manufacturing the scaffold, the different manufacturing methods have their precision limitations. If the parameters of structure design are not within the precision scope, the scaffold will not be completed [26, 27]. Therefore, there were some differences between the scaffold and the natural osteochondral complex. Figure 6A shows the resulting subchondral bone scaffold constructed by stereolithography using resin, based on a previous study [26]. Figure 6B shows the final biomimetic osteochondral scaffold fabricated by gel casting based on rapid prototyping.

**Table 2:** Comparative analysis of morphological characteristics of a natural osteochondral complex and the proposed scaffold

	Natural osteochondral complex	Osteochondral scaffold
Phases	Bone, cartilage, and transitional region	Bone, cartilage and transitional region
Porosity of transitional region (%)	20	30
Porosity of bone phase (%)	60	65
Transitional region		
Defects	Exist	Exist
Connected structure	Imbedding interlocking	Imbedding interlocking
Hole area (%)	7	10
Protrusion width (μm)	51	400
Protrusion width (%)	85	35

## Discussion

The aim and ultimate usefulness of tissue engineering is to create engineered grafts that can interact with their environment in order to provide functionalities that are comparable to the original replaced tissue; the graft must also be integrated by the host [28]. Based on these principles, many bi-phasic scaffolds have been designed and built, and were composed of a cartilage phase and a subchondral bone phase [29–32]. Several scientists have explored different osteochondral composite materials and engineered tissues using a variety of approaches [33–35]. Most of their designs were computer-aided and based on the recognition of the different requirements of cartilage and bone regeneration in osteochondral defects. Sherwood et al. [9] developed a unique, heterogeneous osteochondral scaffold using the TheriForm™ 3D printing process. The material composition, porosity, micro-architecture, and mechanical properties varied throughout the scaffold structure. Woodfield et al. [36] presented and characterized a fiber deposition technique for producing 3D scaffolds. This technique allowed the researchers to design the desired scaffold characteristics layer by layer by accurately controlling the deposition of molten co-polymer fibers. Tampieri et al. [31] used a biologically inspired mineralization process to develop composite osteochondral scaffolds that were organized as different integrated layers with biomimetic features for articular cartilage and subchondral bone. However, none of these previous studies dealt with the concept of the cartilage-bone interface and its micro-architecture and conjunctions. The results of the present study revealed the micro-architecture of this interface, allowing the eventual development of new materials that could mimic this interface and allow for a better integration into the remaining bone.

It has been suggested that the CCZ represents a zone of transitional stiffness and decreases the workload stress at the cartilage-bone interface [37, 38]. Indeed, previous studies [37, 38] showed that the CCZ component, the wavy shape, and the presence of conical connections can effectively reduce the stress on the cartilage and subchondral bone plate, playing a role in the protection of cartilage. It has been suggested to bond cartilage and bone firmly together by anchoring the collagen fibers of hyaline cartilage [39]. Norrdin et al. [40] discovered in horses that increased irregularity of the cartilage-bone interface might reflect increased remodeling during growth, and that the irregularity of the CCZ is affected by the position and thickness of cartilage. Wang [14] reported that both the CCZ and subchondral bone plate contained calcium salts. Hence, the separation of the subchondral bone plate from the CCZ by physical methods is difficult, which may pose a problem for the development of novel engineered grafts.

Lane et al. [41] discovered that very few blood vessels penetrate the subchondral bone plate and end in an open tip; vascularity and the total number of vessels with open ends vary with age and location within the joint. In the pathological process of osteoarthritis, blood vessels may invade beyond the tidemark [42]. Oegema and Thompson have reported that exercise can alter the blood vessel numbers in mildly exercised canines [43]. The sequence of events leading to the replacement of some cartilage tissue by bone depends upon blood vessels being brought into the cartilage via these canals. Based on this data, building a bone scaffolds and cartilage scaffolds separately and sticking them together or separating the two phases completely by a sealing coat might not be the optimal way to regenerate the osteochondral complex. Some scattered defects should be designed to mimic the natural defects that are crucial for blood vessel invasion and nutritional supply. The results of the present study revealed the presence of these defects at the micro-architectural level, and showed that it is possible to design a material containing these “planned defects”.

Based on the cognition of natural osteochondral complexes and previous studies [4, 5, 7, 8, 22–26], an ideal biomimetic osteochondral scaffold should include at least three phases: the cartilage scaffold, the bone scaffold, and the transitional phase between them. Structures should be included in the design of the transitional phase to prevent the delamination of the cartilage phase and the invasion by blood vessels. Passageways for progenitor cell migration should be taken into consideration. However, building an osteochondral scaffold to mimic the natural osteochondral complex is beyond the limits of current technologies [44]. Nevertheless, the method used in the present study and using ceramic stereolithography [26] has been shown to be highly accurate and biologically safe to construct hard tissues with delicate structure. Despite the fact that multiple methods have not been compared together within the same study, results strongly suggest that this method [26] is more precise and more suitable for osteochondral plug fabrication than the previously described techniques [33]. Indeed, Schaefer et al. [33] bioengineered tissues using polyglycolic acid meshes for the cartilage part and a blend of poly-lactic-co-glycolic acid and polyethylene glycol for the bone part, with articular chondrocytes for the cartilage part and periostal cells for the bone part; their results showed that osteochondral tissue composites were successfully obtained. Nevertheless, their method requires the use of two different scaffolds that are combined to obtain the entire graft, while the method presented here uses a single step to create the scaffold. Other methods used a number of different materials (such as β-TCP ceramics and calcium pyrophosphate) but these methods result in scaffolds that are uniform in structural characteristics, without transition between the different zones observed in native bones [26], while the lithography process used in the present study allows the construction of a plug with different structural characteristics. Furthermore, some studies explored the use of bioreactors to populate the scaffolds [34, 35], but the aim of the present study was to examine the fine details of the transitional zone in order to create an osteochondral scaffold that is, as much as possible, similar to the native bone. Additional studies are still necessary to refine the construction techniques of osteochondral plugs and to determine their properties in vivo.

The present study is not without limitations. First, results were not analyzed in terms of weight-bearing/non-weight-bearing regions of the knees, and future studies should address this issue. Results were obtained from cadavers and samples may have suffered from some deterioration. However, it might be impossible to obtain intact fresh non-cadaver knees since patients undergoing knee resection usually does so for good pathological reasons that would affect the results. The aim of the present study was only to provide a morphological analysis of the transitional zone in order to build a biomimetic scaffold, and no quantitative analysis was performed. Finally, the constructed scaffold was not tested in vivo. This will be the focus of our future studies.

The present study is the first part of a systematic research on osteochondral tissue regeneration. This study was only a preliminary study that observed the microarchitecture of the transition zone and that examined if a suitable material could be engineered from these observations. Nevertheless, this material needs to be tested in vivo and will probably require a number of adjustments before being used for treating patients. In addition, only one manufacturing process was used to create the prototype, and the optimal process needs to be defined. Efforts will be made to use these results to generate materials that could be used to repair damaged knees and to treat osteoarthritis.

## Conclusion

Results suggest that the subchondral bone plate is not an intact plate between the cartilage and bone cavity, and that some scattered defects exist that allow blood vessel invasion and nutritional supply. This characteristic was used to design an osteochondral scaffold. This could be implemented to construct an osteochondral complex that is similar to native bones.

## Abbreviations

micro-CT: micro-computed tomography
SEM: scanning electron microscopy
3D: three-dimensional
CCZ: calcified cartilage zone
VOI: volume of interest
BMD: bone mineral density
TMD: tissue mineral density
BMC: bone mineral content
TMC: tissue mineral content
BVF: bone volume fraction
